# Assessment of graduate public health education in Nepal and perceived needs of faculty and students

**DOI:** 10.1186/1478-4491-11-16

**Published:** 2013-04-26

**Authors:** Agya Mahat, Stephen A Bezruchka, Virginia Gonzales, Frederick A Connell

**Affiliations:** 1 Department of Global Health, School of Public Health, University of Washington, Seattle, WA, USA; 2 Department of Health Services, School of Public Health, University of Washington, Seattle, WA, USA

**Keywords:** Public health, Graduate public health education, Nepal, Collaboration, Global health, E-learning

## Abstract

**Background:**

Despite the large body of evidence suggesting that effective public health infrastructure is vital to improving the health status of populations, many universities in developing countries offer minimal opportunities for graduate training in public health. In Nepal, for example, only two institutions currently offer a graduate public health degree. Both institutions confer only a general Masters in Public Health (MPH), and together produce 30 graduates per year. The objective of this assessment was to identify challenges in graduate public health education in Nepal, and explore ways to address these challenges.

**Methods:**

The assessment included in-person school visits and data collection through semi-structured in-depth interviews with primary stakeholders of Nepal’s public health academic sector. The 72 participants included faculty, students, alumni, and leaders of institutions that offered MPH programs, and the leadership of one government-funded institution that is currently developing an MPH program. Data were analyzed through content analysis to identify major themes.

**Results:**

Six themes characterizing the challenges of expanding and improving graduate public health training were identified: 1) a shortage of trained public health faculty, with consequent reliance on the internet to compensate for inadequate teaching resources; 2) teaching/learning cultures and bureaucratic traditions that are not optimal for graduate education; 3) within-institution dominance of clinical medicine over public health; 4) a desire for practice–oriented, contextually relevant training opportunities; 5) a demand for degree options in public health specialties (for example, epidemiology); and 6) a strong interest in international academic collaboration.

**Conclusion:**

Despite an enormous need for trained public health professionals, Nepal’s educational institutions face barriers to developing effective graduate programs. Overcoming these barriers will require: 1) increasing the investment in public health education and 2) improving the academic environment of educational institutions. Long term, committed academic collaborations with international universities may be a realistic way to: 1) redress immediate inadequacies in resources, including teachers; 2) encourage learning environments that promote inquiry, creativity, problem-solving, and critical thinking; and 3) support development of the in-country capacity of local institutions to produce a cadre of competent, well-trained public health practitioners, researchers, teachers, and leaders.

## Background

Nepal is one of the world’s poorest countries with a multi-ethnic population of approximately 29 million. According to the World Bank, 82% of the population live in rural areas and 25.2% live below the national poverty line [1]. In the past two decades, Nepal has made significant improvement in public health indicators. For example, from 1990 to 2008, Nepal’s under-five mortality rate (U5MR) decreased from 137 to 52 per 1000 live births [2], and maternal mortality ratio decreased from 870 to 380 per 100,000 live births [3], making it one of the few countries on track to meet the targets of the Millennium Development Goals in reducing child and maternal mortality.

Despite these gains, Nepal’s maternal health remains the poorest in South Asia [3]. Furthermore, half of the population aged less than 5 years old have stunting and anemia, 39% are underweight, and neonatal mortality accounts for more than half of U5MR [4]. The wide inequalities in health indicators within the country, which are related to geographic location, gender, caste, and ethnicity, are especially concerning. The life expectancy at birth ranges from 71 years in the city of Bhaktapur to 44 years in the remote district of Mugu [5]; low-caste Dalits have U5MR of 90 deaths per 1000 live births, almost twice the national average; and only 14% of deliveries in rural areas are assisted by a skilled birth attendant, compared with 51% of deliveries in urban areas [4].

Although the 18 accredited medical schools in Nepal [6] produce a considerable number of doctors every year, the ratio of 2 physicians per 10,000 of the population is far below the global ratio of 14 physicians per 10,000 [7]. According to the Nepal Health Sector Programme Implementation Plan II (NHSP-IP2), only 4.34% of the total number of healthcare providers are doctors, and even with this number, one-third of the positions for doctors and nurses are unfilled. Moreover, in most rural areas, deployment and retention of all categories of health personnel remains a persistent challenge [8]. This trend is likely to continue, as a recent survey of career intentions of medical students in Nepal showed that 88% of medical students were likely to practice in urban centers [9].

Nepal has an extensive and rationally designed government-run system that integrates community-based primary health care with hospital and specialty care throughout the country [10]. This system, which is responsible for the conduct of preventive and categorical public health programs, such as family planning, immunizations, and nutritional programs, relies heavily on female community health volunteers (FCHVs) and other para-professional workers to provide healthcare delivery to the rural population. The FCHVs are the first point of contact to the health system, and the patients are then referred to the sub-health posts or health posts, and from then on to primary health centers, to district, zonal, and regional hospitals, and finally to specialty tertiary care centers.

Despite the need to implement, manage, and improve the healthcare system, there is a critical shortage of public health experts in Nepal [8]. Most Nepalese with public health training have been educated abroad in western countries or in neighboring countries such as India, Bangladesh, and Thailand. Many of these individuals will either work overseas or, if they stay in Nepal, will work for non-governmental organizations (NGOs) and international non-governmental organizations (INGOs), rather than in the government. In the absence of sufficient local public health expertise, there is a persistent dependence on international consultants to manage public health issues, often leading to inequitable, expensive, and uncoordinated development efforts.

A community-based approach to medical training has always been an integral part of the Nepalese medical-school curriculum [11,12]; however, the inability of Nepali health professionals to efficiently deal with the recent cholera epidemic in western Nepal, which claimed 301 lives, is an example of the inadequacy of public health training in the country [13,14]. There has been little investment into research on epidemiology, health-information systems, or management [11] and, in contrast to medical education, public health education has received relatively little attention or investment. A Masters in Public Health (MPH) is the only available graduate public health degree program in Nepal, and is offered in only two medical institutions, which together produce a total of thirty graduates a year. In Nepal, as in most countries, the MPH is primarily a degree for professional practice. Nevertheless, because of the paucity of opportunities for higher education in public health, it is likely that MPH graduates may also work in research and academic settings.

In 1991, the Institute of Medicine (IOM) at Tribhuvan University (TU) in Kathmandu introduced a 2-year MSc in Public Health (MScPH) program, enrolling three students. Since then, the program has undergone several changes, including re-naming the program degree to MPH. The general objective of the program is to ‘train skilled public health specialists with management, research and advocacy capabilities to meet national and international demand on public health’ [15]. At present, the Department of Community Medicine and Family Health at the IOM offers an 18-month full-time MPH program for 20 students every year.

The B. P. Koirala Institute of Health Sciences (BPKIHS), located in Dharan in the eastern region of Nepal, is the country’s only institution to include a residency program in Community Medicine for physicians. In 2005, BPKIHS started offering a 2-year, full-time MPH program through its School of Public Health. The vision for this program is to reduce the burden of disease, injury, and disability through the development of social policies, healthy environments, effective infrastructure, equitable healthcare systems, and skilled human resources, with the work to be guided by ‘social justice, interdisciplinary collaboration, a population-based focus, and a primary emphasis on prevention’ [16].

Patan Academy of Health Sciences (PAHS) is a new medical institute that plans to offer an MPH program in the near future. One of the contexts for its establishment was the continued need for external consultants to manage ‘problems or agenda of intellectual nature’, because of the absence of indigenous intellectual capital in health sciences in Nepal [17]. The mission of the institute is the ‘sustained improvement of the health of the people of Nepal, especially those who are poor and living in rural areas, through innovation, equity, excellence and love in education, service and research’ [18].

BPKIHS and PAHS are autonomous institutions, supported by the government and affiliated with the Ministry of Health, whereas IOM is a part of a public university run by the government and affiliated with the Ministry of Education.

There are very few studies of public health education in South Asia. In 2000, there were only eight schools of public health in the region [19]. The Calcutta Declaration of the Regional Conference on Public Health in South-East Asia in 1999 emphasized the need for strengthening and reforming public health training programs for capacity building of the public health workforce. One of the specific recommendations was to ‘strengthen and reform public health education and training, and research, as supported by the networking of institutions and the use of information technology, for improving human resources development’ [20]. However, most graduate public health programs in South Asia were offered by medical schools and did not have much effect on public health because of ‘neglect, assignment of lowest priority, low prestige, poor quality of staff and inadequate facilities’ [21,22].

The objective of this study was to identify challenges to implementing effective graduate public health education in Nepal and to explore ways to address these challenges. The findings of this assessment can provide a useful reference for current and future schools of public health in other developing countries similar to Nepal.

## Methods

### Ethics approval

The University of Washington Ethical Review Board granted an Institutional Review Board waiver to this study.

### Participants and data collection

Data for this assessment were derived from individual and group interviews with primary stakeholders and from on-site visits to the schools of public health offering graduate education in Nepal. The primary stakeholders of the Nepalese public health academic sector were identified as public health faculty, students, leadership, and alumni of IOM and BPKIHS, and the leadership of PAHS.

The eligibility criteria for participants were: enrolled students and past graduates of the MPH program at BPKIHS and IOM, and public health faculty and leadership of the departments and institutions offering graduate public health programs during the period of data collection. The Community Medicine residents in one institution were included as participants (students) as they shared one year of the academic MPH program with the MPH students. The leadership of PAHS was included as participants (leadership) as it was involved in planning an MPH program to begin in the near future. The head of the information technology department at one of the institutions was also included as a leadership participant because our e-learning queries were referred to him by the institution’s leadership.

Permission for this assessment was sought once we apprised the public health department chairs and school chiefs of BPKIHS, IOM, and PAHS of the objectives of the study. Sampling of the participants was based on their availability and willingness to talk about their perspective on graduate public health education in Nepal. A snowball sampling technique was used to contact participants in the MPH-alumni category. Contact with the stakeholders was made through email correspondences, telephone calls, and in-person meetings to schedule in-depth interviews and visits to the three institutions in Dharan, Kathmandu, and Patan. A member of the research team spent 9 weeks (August/September and November/December 2010) collecting data in Dharan, Kathmandu, and Patan. In addition, a literature review was conducted to gather information on graduate programs in public health in Nepal.

Individual in-depth in-person interviews were conducted with most of the participants. Individual and group interviews (in groups of two to six) were conducted with students, depending on their availability and interest. A total of 72 participants were interviewed in 49 sessions, which covered more than 50% of the faculty and students enrolled during the period of data collection. The details of the participants are given in Table 1.

**Table 1:** Study participants

**Institutions**	**Faculty**	**Students**	**Institutional leaders**	**Alumni**
IOM	9	21 (8 sessions)	3	5
BPKIHS	10	16 (6 sessions)	2	4
PAHS			2	
Total	19	37	7	9
Grand total	72 participant interviews in 49 sessions			

All interviews with the leadership and faculty and most of the interviews with students occurred within the campus premises in a variety of private settings as preferred by the participants (offices, staff-rooms, classrooms, cafeterias and residence halls). Other interviews took place at the workplaces of the students and alumni, except for an interview with one alumnus that took place in a cafe. Depending on the preference of the participants, most of the interviews were conducted in Nepali and a few in English. All participants gave verbal consent for the interviews. Most of the interviews were digitally recorded; five interview sessions were not recorded because of technical issues or participant concerns.

A semi-structured open-ended questionnaire (see Additional file 1) was used to gather information regarding the participants’ perception about graduate public health education in Nepal, with prompts for key issues such as the strengths and challenges of the programs, ideas about ways to solve the challenges and their views on collaborations, use of internet and e-learning. The most common emerging themes identified from earlier interview sessions were also added as key prompts in later interviews to achieve theoretical saturation.

The interviews were transcribed into English using a word-processing program (Word; Microsoft Corp., Redmond, WA, USA) and checked several times for accuracy. Emerging themes identified through content analysis were tabulated (Excel; Microsoft) along with the corresponding data and participant characteristics. The research team discussed and agreed on all salient themes.

## Results

In general, participants cited the strengths of the current MPH programs as being the country-focused curriculum and mandatory field requirements, which give students a practical understanding of Nepal’s public health system and challenges. A competitive entrance examination, an annual graduation rate of over 95%, and the high demand for MPH graduates suggests a high level of competency of graduates. In addition to these strengths, six themes characterizing the challenges of graduate public health education were identified. In this section, we describe these six themes, and for each theme, we include direct quotes from respondents to help illustrate the issues they identified.

### Shortage of adequately trained faculty and education resources

Most stakeholders expressed the concern that there is a critical lack of available, well-trained public health faculty to teach graduate students. Currently, the schools of public health in Nepal have no faculty with a Doctor of Public Health (DrPH) degree, and very few faculty with a PhD or a specialized MPH degree (from institutions outside of Nepal). Recent graduates with a general MPH, or an MD in Community Medicine, who generally have very limited experience in public health practice or research, are hired as faculty and assigned one or more subjects to teach. The shortage is most apparent in areas of health economics, management, policy, medical anthropology, qualitative research, environmental and occupational health, nutrition, and biostatistics. Because of these unfilled faculty vacancies, a single instructor may have to teach multiple subjects in a variety of disciplines, often with no or minimal formal education in these topics. In addition, temporary faculty from non-public health disciplines, such as economics or management, may be hired to teach, and it is often left to the students to relate these concepts to public health issues.

*‘…we are public health students but there isn’t any public health expert teacher here.’* (Student)

*‘… [Even though] I don’t have any [special] training as such in epidemiology, [in general] I teach everything - field epidemiology, molecular epidemiology, medical epidemiology.’* (Faculty member)

Furthermore, there is lack of adequate physical and informational resources for faculty and students in MPH programs. The campus libraries are short of up-to-date public health books and journals. Even for in-country reports, students may need to make trips to the Ministry of Health and Population or other relevant institutions to obtain a copy.

*‘If we compile [the personal] books of our cohort only - without duplicating- we have twice the number [of books] than the public health library.’ *(Student)

Statistical software (for example, EpiInfo and SPSS), is used for quantitative data analysis, but training in these programs is inadequate for both students and faculty. Each school had only one faculty member well versed in statistical software who could help students with their data analyses. Students and faculty rely on the internet to compensate for the shortcomings in instruction, such as the shortage of books, journals, and other educational resources.

*‘… Google is [our] best professor.’* (Student)

The non-search engine use of the internet ranges from literature searching to viewing videos of lectures on YouTube. There is some access to online literature through the Health Internetwork Access to Research Initiative (HINARI), a facility provided by the WHO, which allows access to many international journals free of cost. Although most faculty and students own a personal laptop, the institutions have been unable to provide them with an adequate number of computers, consistent high-speed internet connections, printing facilities, or software licenses.

Despite extensive use of the internet, only a few faculty members and students had heard of e-learning, and fewer had taken formal web-based courses in public health. Those students and alumni who have had some exposure to e-learning were optimistic about the effectiveness of such resources as supplements to classroom education, whereas the leadership was generally cautious because of unfamiliarity with e-learning, and concerns about the technological and financial capacity of their institutions to implement such programs.

### Within-institution dominance of clinical medicine over public health

Situated within medical institutions, public health receives a low priority in terms of funding and investment, which is exacerbated by the fact that such departments do not generate financial profits from clinical services. In the view of both students and faculty, clinical departments are deemed more prestigious. For example, even though students without a medical background may have more public health experience and perform better in examinations than their physician classmates, they receive less recognition and mentorship and fewer benefits (for example, they may have poorer accommodation facility) than physicians taking the same classes. Faculty salary and promotion criteria favor physicians, who occupy the most influential positions in these institutions. Even though a physician faculty member in a school of public health may not actually perform clinical duties, they will often make 150% to 170% of the salary of a non-physician colleague. This has led to low morale among non-physician faculty and students, and makes an academic career in public health less attractive for non-physicians.

*‘The most neglected section of this institute is public health. In that, I think the most neglected degree is MPH.’* (Student)

*‘What the non-clinician says is underestimated, they are not taken seriously.’* (Alumni)

*‘MPH can be done by anyone. Because of my medical background I know more about disease.’* (Faculty Member)

*‘Their background influences their way of teaching, perception and explanation … they always relate medicine with public health.’* (Student, about clinicians as public health faculty)

At least 50% of the public health faculty in the institutions are physicians, whereas the majority of students come from diverse, non-physician backgrounds. Both students and alumni noted that the physician faculty members with less experience in public health practice emphasize pathobiology and medical care rather than the broader population-based focus that public health demands, such as emphasis on epidemiology, policy, and management. Institutional libraries cater predominantly to the need of the medical students, and the student assignments, policies, and evaluation criteria generally follow a medical-school model. Most non-physician participants believed that separation of public health programs from medical schools and departments of community medicine would yield better results for public health education.

### Teaching and learning cultures and bureaucratic traditions

Generally, systems are not in place for either student or peer evaluation of teaching, nor are there concrete rewards for good teaching. Student-teacher interactions are often inhibited by adherence to traditional roles, which discourages disagreement with, or questioning of instructors.

*‘The relationship between teachers and students is very strict…you have to maintain the distance.’* (Student)

A common practice in graduate public health education in Nepal is to assign certain topics in the curriculum for students to teach to the class in order to encourage active participatory learning while the teachers act as facilitators. However, according to the students and alumni, constructive feedback on student presentations and even satisfactory clarifications to student queries are not always available, for reasons such as inadequacies in faculty expertise and presumed reluctance to give students more information.

Faculty involvement in research and emphasis on publication is uncommon, because there are minimal incentives, including promotion criteria, from the institutions to conduct and publish research in peer-reviewed journals. The research opportunities within the hospitals and affiliated health facilities of the institutions are untapped because of the absence of interdisciplinary collaboration and the prevalent notion that public-health research has to be community-based. In addition, community-based research may not be recognized as rigorous or scientific.

*‘I haven’t published a paper after my PhD [because] here research is not taken [to be important]. To do research we need some minimum [resources].*’ (Faculty member)

*‘To empower and motivate me for research, for promotion, I need a PhD… You see the environment is different in developing countries. Everyone here wants a timely promotion … that is a big factor for motivation’.* (Faculty member)

*‘Public health does not only mean going out to the community… I was told ‘What good will a hospital based research do for public health?”* (Alumni)

The public health departments/schools are required to go through a long bureaucratic process to get approval from the higher authorities of the universities to make changes in the departmental activities. Thus, hiring new faculty members can be a long process. Because both of the institutions that house a public health program are government-run or government-supported, frequent changes in government are often followed by political appointments in academic institutions. A faculty can also be given a political appointment in non-academic areas without taking into account the effect it will have on the students. Senior public health experts within Nepal and public health specialists trained in other countries do not wish to join the public service at a junior-level position, which is usually the only point of entry into the Nepalese public academic service.

*‘I think people will work if we can create the [right] environment.’* (Leadership)

The public academic institutions in Nepal follow a system of employment that does not take the quality or influence of a faculty member’s teaching, research, or service into account for job retention and promotion. Policies regarding the hiring, promotion and dismissal of permanent faculty, as well as the issue of inadequate resources and the absence of merit-based advancement, aggravate the lack of passion within the faculty for teaching public health, and the inability of the institutions to retain faculty in public health. Even the regularity of classes depends on the ability of the students to exert pressure on the faculty. It is common practice for students to take extreme measures such as demonstrations and strikes before their concerns are taken seriously.

*‘Their attitude is that it is just a job to earn a living, not actually to run this program. When such faculty trains students, most of them will turn out to be the same too.’* (Student)

### Need for practice-oriented and contextually relevant public health training

The institutions award the MPH degree based on classroom attendance and student performance in formal examinations. A major area of dissatisfaction among students and alumni is the predominantly theory-driven classroom education, with little emphasis on applicability. Most classes focus on presenting only descriptive information about existing health systems and theories, instead of encouraging discussions on practical approaches, controversial views, or imaginative thinking. Some senior faculty members justify the traditional system of education and evaluation because of the belief that application is only possible after mastering the theory. However, according to alumni, there is a large gap between the public health theories and practice, and consequently they often learn the skills required in professional settings through frequent trial-and-error attempts. Although the students find the exit examinations useful, some express discontentment because the evaluation of their performance in examinations is based exclusively on their ability to learn by rote and to agree with the teacher’s opinions.

*‘[In the exam] we were asked, ‘what are the Millennium Development Goals?’ …. The questions should have been more like, ‘which of the MDGs are we likely or not likely to meet and why?’’* (Student)

*‘The main frustration for me is that the theory we are learning, I don’t know where I am going to apply that theory.’* (Student)

Some leadership respondents and alumni agree the current programs have been unable to impart creativity or analytical and leadership skills in graduates. An obvious illustration of the gaps in education, they cite, is the absence of public debates over MOHP health policies, lack of change in public systems, and the continued need for expatriates in designing health policies and managing epidemics and disasters in Nepal.

*‘The curriculum should give that outlook, vision… Otherwise [we accept] it has always been like this in the system.’* (Alumni)

### Demand for more degree options and specialized public health training

Student and alumni expressed a strong desire and need for specialized and advanced programs in public health, such as specialized MPH, PhD, and DrPH degrees. Although the existing programs are generally designed for ‘generalist’ graduates to work at district level, they do not adequately prepare the graduates for work in ‘specialist’ public health roles, such as policy, teaching, or research, which require more expertise in a particular area such as quantitative and qualitative methods, management, and health economics. Faculty members with a generalist MPH degree feel inadequately trained to teach graduate students, and the alumni working at MOHP assert that there is a reliance on specialists working in NGOs for assistance in areas that require their expertise, because of the lack of public health specialists in the Ministry.

*‘Wherever you go, they will ask you what is your specialty… generalists can do everything but nothing is in-depth.’* (Alumni)

### Desire for international academic collaborations

Participants believed that academic collaborations with international universities and research institutions could help overcome to a large extent the inadequacies in resources. This should include not only teaching, but faculty and institutional support and building capacity through joint research projects and PhD programs. One institution believes that collaborations are essential to incorporate elements of western education such as a curriculum with emphasis on leadership, critical thinking, and analytical skills, which the public health education in Nepal has lacked up to now.

*‘What we want [is] the expertise… their perception, scientific method, analytical skills and techniques… How can we give our students that opportunity to learn something they will be able to exercise in a real sense?’* (Leadership, on expectations from international partners)

The schools/departments of public health received extensive support from different organizations in the initial years of their establishment. The benefits from collaborations with several international organizations and universities have included support for infrastructure; for resources such as computers, literature access and assistance in curriculum design; and for faculty development through scholarships for MPH, M Phil and PhD degrees, workshops and short-term training, joint research projects, and faculty and student exchange programs. However, the schools still struggle to run their existing programs effectively and are unable to meet the demand for more or expanded programs. Some participants believe that local institutions could have benefited more from previous collaborations had it not been for the inequities that existed in those partnerships, such as dominance of the international partner in resource access and decision-making. The desire now is to form partnerships based on equity and mutual benefit instead of the designated roles of the dominant ‘giver’ and the subordinate ‘receiver’.

## Discussion

The critical state of the education sector is one of the many consequences of the political instability in Nepal, which has persisted for over two decades. Furthermore, external development partners have been far more generous in supporting government and NGO public health programs than they have been in supporting development of human resources.

### Barriers

Despite the enormous need for an effective public health workforce in Nepal, the educational institutions face multiple barriers to developing high-quality graduate-level academic programs. The fundamental challenge is the critical shortage of well-trained faculty with advanced and specialized degrees and significant work experience. In addition, institutional policies create disincentives for hiring and retaining faculty. For example, the differential status and salary between public health and clinical faculty, limited opportunities for faculty development, absence of accountability for teaching performance, and time in rank-based (as opposed to merit-based) promotion criteria inhibit the enthusiasm, professional growth, and commitment of current or potential faculty who might be considering a career in academic public health. Finally, issues such as inadequate reference materials in the library, inadequate internet and computing resources, and the unavailability of basic office supplies also contribute to the difficulties faced by students and faculty.

### Curriculum

Based on our assessment, the Nepal-focused, generalist MPH curriculum, with its emphasis on self-learning and community-based thesis research, gives students a practical understanding of health systems and prepares them to work as public health practitioners in districts. However, it does not prepare them adequately to work as public health academicians, researchers, and leaders. Such positions require the availability of graduate programs focusing on specialized areas such as epidemiology, biostatistics, health policy, and management. In addition, accredited continuing-education programs on public health are also required for graduates and professionals to strengthen and improve their knowledge and skills and to engage in a culture of lifelong learning. We did not find any evidence that these programs currently exist in Nepal. Advanced programs (PhD and DrPH) are also needed to meet the demand for faculty expertise and leadership skills. At the current time, however, the development of stronger MPH-level programs is a more realistic strategy for the country, particularly given the severe shortage of faculty who would be qualified to teach in doctoral programs.

### Quality of teaching

We found a contradiction between the perception by students and faculty of the quality of teaching in schools of public health. Although a majority of students and alumni believed that most instructors lacked adequate expertise and teaching skills, only a few instructors admitted having shortcomings in teaching skills. For the most part, teaching public health was not regarded by the faculty as a challenging profession. Some faculty emphasized the value of self-learning by graduate students under faculty guidance, but students were not uniform in their satisfaction with the guidance they received. Students preferred class activities that closely resembled their future professional duties, and evaluation methods that reflect their level of understanding of public health issues instead of being a mere test of memory. However, educational traditions in South Asia (for example, the authoritarian, hierarchal relationships between faculty and students and the emphasis on rote learning) are not well suited to public health education, which, ideally, should encourage open discourse, interdisciplinary cooperation, inquisitiveness, and creative problem-solving. Our findings suggest that most of the faculty members are not adequately trained to be effective teachers and that the conventional theory-driven teaching approach has failed to impart creativity, leadership abilities, and analytical and problem-solving skills.

Although student and alumni evaluations of academic programs are commonly used in western countries, and have provided valuable input to improve the curriculum and faculty performance [23-25], the absence of official student evaluations of faculty performance for these programs shows an indifference towards the quality of teaching and a disregard for student views in the academic culture of Nepal. An additional cause of student dissatisfaction with teaching was that, financially lucrative opportunities, when available, often took priority over teaching for some instructors. Overall, the perceived low priority that the faculty gave teaching and research was detrimental to the students’ enthusiasm for, and mastery of, public health skills and knowledge.

### Academic environment

This study found considerable agreement about the inequitable power and privilege afforded to medical compared with non-medical faculty, especially in the areas of salary, promotion criteria, opportunities for professional development, and leadership assignments. Dominance of physicians within schools of public health may be one of the major reasons behind the critical shortage of teachers in areas such as biostatistics, health economics, environmental health, social sciences, and health management, which are all disciplines that do not fall into the conventional domains of a medical school curriculum. Although Community Medicine is an integral part of Nepalese medical school curricula, we found that a synergistic relationship between departments of public health and clinical sciences has not been well implemented in actual practice. Furthermore, the prevalent notion that public health research has to be ‘community-based’ has discouraged interdisciplinary research within the institutions themselves. Schools of public health in Nepal reside in educational institutions that also run hospitals, affiliated specialized health centers, and peripheral health facilities, all of which are engaged in the treatment and prevention of conditions of global public health importance, such as infectious and chronic diseases, trauma, maternal and child health problems, and mental illness. Collaboration between public health and clinical faculty (and students) could be a natural and effective means of conducting research and implementing programs to combat these problems.

### Looking forward

The literature suggests that initiation of change in academic cultures should come from leadership, such as deans and departmental chairs, and that the support of senior faculty for these changes is crucial [26,27]. However, our study shows a lack of unity among public health faculty in Nepal and considerable caution regarding innovation and change.

The findings of this assessment are not unique to Nepal. The literature on public health education in developing countries in Africa cites similar challenges: faculty shortages, low morale, retention issues, inadequate physical infrastructure, and variable commitment of the faculty, which diminishes their effectiveness in teaching [28,29]. A lack of training in leadership and independent thinking skills, and the suboptimal quality of programs have been reported as challenges for public health education by others in South Asia [22,30]. In the past, public health education in the US and UK also faced similar issues: low prestige, faculty shortages, inadequate resources, deficiencies in computing resources, poor teaching, concerns about the lack of specialized and doctoral level training, and inadequate integration of theory and practice [23,31,32]. The *Lancet Commission Report on Education of Health Professionals for the 21st Century* states that professional education has not kept pace with the challenges in public health globally ‘because of fragmented, out-dated, and static curricula’ and recommends transformative competency-based curricula that subsume focus on analytical abilities, leadership and management capabilities, communication skills, and a culture of critical inquiry [33].

Historical evidence suggests that the rift between medicine and public health widens when the two are separated from each other [32,34], and poor countries, in particular, cannot afford a separation of the two fields. The literature recommends flexible and/or equal recognition of diverse education and professional backgrounds to meet the increasing demands posed by emerging public-health issues [35]. In the UK, following the replacement of ‘Faculty of Community Medicine’ with ‘Faculty of Public Health,’ uniform certification procedures were introduced for all public health professionals regardless of previous educational backgrounds in order to recognize those with versus those without specialist public health knowledge, rather than division into physicians versus non-physicians [35-37]. Although Nepal was one of the signatories of the South-East Asia Public Health Initiative, which emphasized that public health is multidisciplinary [38], equal recognition of (non-physician) public health professionals does not exist in practice.

In other countries, the introduction of specialist and doctorate public health training has occurred over time in response to demand [39-42]. Currently, there is a demand in Nepal for specialist MPH programs and advanced programs such as PhD and DrPH, with a critical shortage of health system-related human resources. Speciality and advanced training is crucial to meet the needs of government programs and academia for expertise in health policy, epidemiology, health economics, and systems development [8]. This need is also illustrated by the findings of the current study: faculty often lack the academic preparation to teach specialized topics; vacant faculty positions remain unfilled for long periods; students are often unable to find faculty with the desired expertise in their area of research interest; and the MOHP commonly depends on help from the non-government (often international) consultants for specialist public health expertise.

### Resources and sustainability

The Rockefeller Foundation Public Health Schools without Walls programs in Africa and Asia demonstrates that high-quality public health training is possible in developing countries, but that this requires substantial external resources [43]. There have been calls for various international agencies to support public health training institutions in developing countries through collaborations [19,34,38,44]. The James P Grant School of Public Health in Bangladesh is a result of such collaboration between the Bangladesh Rural Advancement Committee (BRAC) and multiple international partners [45]. Respondents in this study expressed cautious interest in developing international collaborations to address the inadequacies of existing programs and to develop additional graduate programs in public health, for example, by having faculty from established international schools of public health participate in mentoring programs to encourage teaching/facilitation skills that focus on developing critical thinking, analytic skills, and leadership capability in public health students. Partnerships between schools of public health in wealthy and poor countries could help improve the design of classes and curricula, and could promote research projects and research training. These are activities that could benefit both sides in the short and long term [46-48].

Funding is always a challenge in long-term collaborations, but might be reduced to some extent if the support can build on electronic communication, internet resources, and e-learning. We found considerable interest among students and alumni in e-classes that could complement and/or supplement the existing classes. There was optimism that e-learning resources in classroom settings, using a local faculty to facilitate and integrate the teaching of the web-based material, could be effective. E-learning may be a cost-effective and sustainable approach in public health collaborations between academic institutes, provided the technological requirements are met. Moreover, it can also be used to conduct continuing-education programs. Several academics have reported successful collaborations between schools of public health through e-learning [29,40,49,50]. Evaluations of some e-learning programs have found no difference in learning outcomes between traditional face-to-face and distance learning [51,52]. There is, however, a lack of published literature on the use of e-learning for public-health education in South Asia.

However, it was pointed out by some respondents that the inequalities that existed in some previous partnerships with international institutions have created a sense of distrust and cynicism in some stakeholders about future collaborations. This cultural divide or imbalance in international academic collaborations is not uncommon [53]. Furthermore, critics have pointed out that international partners may have inadequate understanding of, and experience of working within, the local culture and conditions, and that even experts can have minimal relevant field experience, especially in developing countries [32].

A common challenge to international collaboration is sustainability. Previous partnerships in Nepal indicate that sustainable progress in institutional and infrastructural development requires long-term collaboration. Our findings suggest that it may be less challenging to start a new public health program than to maintain and improve an existing one over time.

The lessons learned from academic collaborations in public health in Nepal and elsewhere point to several essential conditions that must be met: 1) first and foremost, addressing the needs of the local institutions; 2) not agreeing to collaborate simply to receive ‘whatever we can get’; 3) clarifying the terms of the partnership in writing; 4) planning for sustainability; and 5) requiring that partnerships be based on mutual trust and equitable control.

### Study limitations

Thematic saturation of qualitative data from a large sample size yielded rich information in this assessment, but several limitations to this study exist. The study participants were the primary stakeholders only in the public health academic institutions in Nepal. The perspective of other stakeholders such as MOHP, local public health agencies, and NGOs may differ from the findings of this assessment. Snowball sampling of alumni might have resulted in findings that do not reflect the perception of all alumni. The researcher who conducted the interviews was a Nepali MPH student at the University of Washington with a medical degree in dental surgery. Her background provided a comfortable rapport with the participants; however, her connection to a political family in Nepal may have affected the responses of some participants. Although we synthesized the data to identify the strongest themes, researcher bias in the analysis is inevitable to some extent because of the past experiences of the authors.

### Recommendations

Based on the finding of this study and the literature cited, we suggest the following recommendations for improving the quality of public health education in Nepal. Although these recommendations are aimed primarily at the issues we identified in Nepal, we expect that they may have relevance to the development and enhancement of schools of public health in other developing countries.

### Create a strong faculty

The leadership of academic institutions should give the highest priority to faculty training and development programs. This will require identifying opportunities (including funding) for faculty pursuing advanced degrees abroad. However, local training in use of essential tools (such as teaching methods, statistical software, research methodology, grant proposals) should also be pursued using both local and visiting experts.

### Align teaching methods to course content and student learning needs

Public health faculty should be encouraged to adopt innovative approaches to teaching that focus on developing critical thinking ability, analytic skills, and leadership traits in their students.

### Promote merit-based criteria for promotion and other faculty rewards

A powerful incentive to improve teaching, as well as to generate enthusiasm for research, is to base promotion, professional travel, and salary on considerations related to productivity and merit.

### Implement student/alumni/peer evaluation of teaching

Regular evaluations of teaching from peers, students, and alumni (and mentors, if possible) is an effective means to continuously improve teaching, update courses, and enhance the overall curriculum.

### Improve physical infrastructure and resources

An adequate number of computers and high-speed internet facilities are essential to provide access to articles and texts, documents, data, e-learning, and internet-based communication (for example, Skype).

### Engage in mutually respectful international collaborations

Collaboration with international schools of public health can enhance teaching and research by mentoring local faculty, developing curricula, conducting joint research projects, seeking external funding, and identifying opportunities for faculty development and exchanges.

### Diversify graduate programs in public health

To meet the demands for more academicians, researchers and public health specialists in Nepal, institutions need to develop specialized and doctoral degree programs in public health, and to promote opportunities for continuing education.

### Utilize e-learning

E-learning can be a cost-effective tool for enhancing the curricula of degree programs for providing continuing education. This will require encouraging use of available e-learning resources and exploring ways to ensure the technological requiremenst are met.

### Promote interdisciplinary research and eliminate medical dominance

The leadership in the academic institutions and MOHP need to work on introducing similar accreditation requirements for public health graduates irrespective of their previous educational backgrounds. This will bring a much-needed multidisciplinary, cross-sector approach to addressing the public health needs of Nepal. Furthermore, within institutions, programs in public health and community medicine should be coordinated, and measures should be taken to address the physician/non-physician discrimination in academia and in government agencies.

### Give more autonomy to schools of public health

Revisions of institutional policies may be necessary to give schools of public health parity with schools of medicine with regard to academic autonomy and access to resources.

### Conduct regular evaluations

Schools of public health should be formally evaluated at appropriate intervals, using both internal and external reviewers. In addition, annual internal reviews should be conducted to assess progress towards institutional goals and objectives.

## Conclusion

There is an urgent need to increase investment in public health education and to improve the academic environment of academic institutions. Nepal is transitioning to a more engaging democratic society and has been successful in making progress towards a range of health goals. Improving the status of in-country public health education is a necessary step in this process to produce a cadre of competent, well-trained public health practitioners, researchers, teachers, and leaders. The qualitative research we have conducted presents some important elements to consider in effecting this change.

## Abbreviations

BPKIHS: BP Koirala Institute of Health Sciences; BRAC: Bangladesh Rehabilitation Assistance Committee; HINARI: Health Internetwork Access to Research Initiative; IHME: Institute for Health Metrics and Evaluation; IOM: Institute of Medicine; JPGSPH: James P Grant School of Public Health; MD: Doctor of medicine; MMR: Maternal mortality ratio; MOHP: Ministry of Health and Population; MPH: Master of Public Health; MScPH: Masters of Science in Public Health; NGO: Non-Governmental Organization; PAHS: Patan Academy of Health Sciences; TU: Tribhuvan University; U5MR: Under-five mortality rate; WHO: World Health Organization.

## Competing interests

The authors declare that they have no competing interests.

## Authors’ contributions

AM participated in the study design, coordinated and collected data, performed data analysis, and drafted the manuscript. FAC conceived the study, participated in its design, supervised the data collection and analysis, contributed to the drafting of the manuscript, and edited the manuscript. SAB participated in the study design, supervised the data collection and analysis, and edited the manuscript. VG participated in the study design, supervised the analysis, and provided suggestions on the draft of the manuscript. All authors read and approved the final manuscript.

## Authors’ information

AM is a Nepali dentist and performed this study as her thesis project for her MPH degree in Global Health at the University of Washington. FAC is the Associate Dean of the School of Public Health and Professor of Health Services, Epidemiology and Paediatrics at the University of Washington, and has taught public health in Nepal. SAB is a senior lecturer in the Department of Global Health and Health Services at the University of Washington, and has spent over 10 years in Nepal working in various health programs and teaching in remote regions. VG is a senior lecturer in the Department of Global Health and Health Services at the University of Washington, and also serves as a senior technical advisor at the International Training and Education Center for Health.

## Supplementary Material

Additional file 1 Questionnaire. Click here for file
