# Supplementary material for: Assessment of graduate public health education in Nepal and perceived needs of faculty and students
Source: Hum Resour Health. 2013 Apr 26;11:16. doi: 10.1186/1478-4491-11-16 (PMC3640966; doi:10.1186/1478-4491-11-16)
Supplement: Additional file 1 — Questionnaire. [file 1478-4491-11-16-S1.pdf]

APPENDIX: QUESTIONNAIRES

(a) Questions for Faculty and Leaderships:

Can you talk about your experience in teaching public health? (probes: previous education, work, duration of teaching, expectations from students)

Can you talk about the delivery of the curriculum, course materials and other resources available for teaching?

Can you tell me about faculty development?

Can you tell me about the strength and weakness of the current program?

What has been the most difficult part in teaching public health in Nepal? How do you deal with it?

If you could change anything about the training, what would it be? (Talk about what would you need to make these changes?)

How do you feel about distance learning classes? (probes: Offering them? Receiving information using this methodology?)

Can you talk about your graduates? (probes: reputation, how do they compare with others?)

Would a partnership with international universities be of value? How so?

Additional questions for Leaderships:

Can you tell me about your experience in developing/running a school of public health/dept. of public health? What are the challenges?

Would you be interested in collaborating with international schools of public health for enhancing the public health training? What form would you prefer that it take (e.g. modular, visiting faculty….)?

(b) Questions for Students

Can you tell me about coming here for an MPH?

Can you talk about the resources available on campus? (probes: faculty hours, library, internet, computers, programs)

Can you talk about the program so far? (probes: challenges, strengths, how program has met expectations so far)

Please tell me about things you would like to see being done differently

Can you tell me about using the internet for assignments?

Please tell me what you think about distance learning classes

(c) Questions for Alumni

Can you tell me about your MPH training?

What were challenges in the training? Talk about how you dealt with it?

Tell me what you liked about the program?

Tell me about graduates from the program. (probes: what do they do after completing the program)

Can you talk about what you would like to see changed in the program.

Can you talk about how these changes might take place?

Please tell me your thoughts on distance learning classes
